# “Asthma can take over your life but having the right support makes that easier to deal with.” Informing research priorities by exploring the barriers and facilitators to asthma control: a qualitative analysis of survey data

**DOI:** 10.1186/s40733-015-0011-5

**Published:** 2015-09-29

**Authors:** Rebecca Normansell, Emma Welsh

**Affiliations:** Population Health Research Institute, St George’s University of London, Cramner Terrace, SW17 0RE UK

**Keywords:** Asthma, Patient participation, Qualitative research, Research prioritisation, Cochrane Airways

## Abstract

**Background:**

Involving patients and the public in research prioritisation is important. Cochrane Airways works with authors to produce systematic reviews of evidence related to chronic airways disease. Cochrane Airways has undertaken activities to identify research priorities, including workshops with stakeholders and consultation with experts. We present the findings of an online survey, designed to align our work with the priorities of people affected by asthma.

**Methods:**

We promoted a survey comprising open-ended questions via social media to people affected by asthma. We compiled the free-text responses and conducted an exploratory thematic analysis to identify important barriers and facilitators to asthma control. We triangulated findings with other research prioritisation activities to produce new review questions.

**Results:**

We received 57 survey responses. Eight main themes emerged, most encompassing both facilitators and barriers: attitudes and knowledge; financial costs; environmental factors and triggers; healthcare systems; lifestyle factors; medication; self-care; and support. Barriers were more frequently mentioned than facilitators and many related to healthcare systems.

**Conclusions:**

These findings offer valuable insights into the challenges faced by individuals affected by asthma in the UK, and possibly further afield. We developed a list of priority reviews based on what was said by people in this survey and at a workshop. This demonstrates the real impact that people affected by asthma have on the research agenda of Cochrane Airways. Over the next 2–3 years we will produce reviews that address some of these questions hopefully leading to health benefits.

## Background

Cochrane is an independent, global network with an aim to make health research accessible, useful and relevant in order to better inform health decisions [1]. Cochrane is organised into a number of groups which focus on disease areas, themes or research methodologies. Cochrane Airways [2], based in London (UK), is one such group. We primarily produce overviews of evidence (known as ‘systematic reviews’ or ‘meta-analyses’) about long-term breathing conditions including asthma, chronic obstructive pulmonary disease (COPD) and bronchiectasis.

Many Cochrane groups have processes by which they attempt to prioritise research topics, which may involve consultation with expert professionals, patients, advocates, and the public [3–5]. There is a growing body of literature exploring patient and public involvement in research [6–8], including scoping reviews [9, 10] and reports describing patient and public involvement in research priority setting for respiratory conditions [11, 12] and more specifically asthma [13].

This paper reports the findings of an online survey of people affected by asthma, which we designed to inform the ongoing prioritisation work of Cochrane Airways [5]. We were particularly interested in exploring perceived barriers and facilitators to effective asthma control as evidence suggests that control remains suboptimal for many people [14]. Poor asthma control is inversely correlated with quality of life [15] and puts patients at higher risk of exacerbations [16]. Reducing exacerbations and improving quality of life are two of the most important goals in asthma management. Therefore, seeking patients’ perspectives on the barriers and facilitators to effective asthma control is important not only for clinicians and health services but for those prioritising research in this area.

### Aim

To inform research priority-setting by conducting a thematic analysis of the views of people affected by asthma.

## Methods

We invited participants with asthma, or affected by asthma, to complete a short voluntary anonymous questionnaire about asthma. EW designed the survey, posted it on Survey Monkey and advertised it alongside an invitation to attend a face-to-face workshop, through the Asthma UK [17] Research and Policy Volunteers Group, the Asthma UK Facebook and Twitter profiles and Cochrane Airways social media and website. For pragmatic reasons, related to the wider ongoing prioritisation exercise, the survey was only available for one month, between 6 August and 5 September 2014. We had no target number of responses.

The five questions allowed an open-ended free-text response and are listed below:

1. What do you do when you have an asthma attack?
2. What problems do you experience in using healthcare services?
3. What are the problems/issues you face in taking your regular asthma medication? (e.g. steroid inhaler)
4. What are the barriers to controlling your asthma?
5. What things help your asthma most?

We compiled the free-text responses verbatim and subjected them to an exploratory thematic analysis [18]. Two researchers (RN and EW) read and re-read the data for familiarity before assigning codes within each question. We resolved coding discrepancies by discussion between researchers before refining the codes to produce higher-order themes, spanning the five questions. We triangulated the emerging themes with research priorities established in the prioritisation workshop, which ran in parallel to the questionnaire. We grouped the themes further to produce broader themes, each encompassing several sub-themes. We constantly revisited the data to confirm and test the credibility of the interpretation. As we did not have any details about respondents, other than that they are affected by asthma, we did not perform a case-based analysis.

**Fig. 1:**
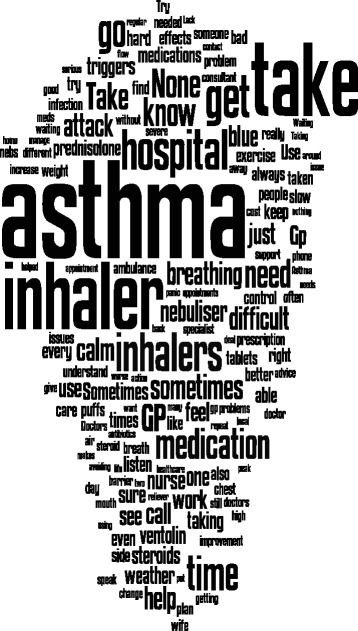
Word-cloud illustrating the relative frequency of different words in the survey responses. From http://www.wordle.net/

We also explored word or code frequency in the questionnaire responses to examine the relative weighting of different concepts. This is displayed pictorially in Fig. 1 by way of a word-cloud.

## Results

We received 57 responses with the majority of people answering all five questions: question 1 answered by 57/57; question 2 answered by 56/57; question 3 answered by 51/57; question 4 answered by 47/57; and question 5 answered by 49/57.

Responses ranged from one word to paragraphs of over 100 words. Although interpretation of the questions varied somewhat between respondents, most codes appeared frequently suggesting substantial agreement in most areas and a likelihood of data saturation. Eight main themes emerged from the data, most encompassing both facilitators and barriers: attitudes and knowledge; financial costs; environmental factors and triggers; healthcare systems; lifestyle factors; medication; self-care; and support. The agreed themes and sub-themes are presented in Table 1 and themes with further exemplar quotes are presented in Table 2.

**Table 1:** Themes and sub-themes

Theme	Sub-themes
Attitudes and knowledge (perceived/experienced)	Healthcare professionals
	Others
Costs	Prescription charges
Environmental factors and triggers	Weather
	Allergens
	Other triggers
Healthcare systems	Timely access to care
	Follow up and continuity
	Communication
	Obtaining medication
Lifestyle factors	Inconvenience/time
	Exercise
	Diet
Medication	Efficacy
	Education
	Side effects
Self-care	Monitoring
	Personalised action plan
	‘Common sense’ measures
	Staying calm/breathing exercises
Support	Healthcare professionals
	Others

**Table 2:** Themes, sub-themes and exemplar quotes

Themes	Sub-themes	Exemplar quotes
Attitudes and knowledge	Healthcare professionals	*“They can sometimes treat you like a waste of space, and from experience I have been left in an A&E* (accident and emergency) *corridor alone after being brought in having an asthma attack wheezing and struggling to breathe”*
		*“I have been having a bad couple of years with my asthma and have become so fed up with the continual disbelief from anyone in healthcare that what I'm reporting is actually happening (even when it happens in front of them!). This is compounded by the fact that every time I talk to someone about it they find a way to say this is all my fault - anxiety, not taking medications or just making it up - when all the evidence proves none of that is true. Asthma = panic attacks is a very outdated and dangerous assumption which seems more rife amongst healthcare professionals now than ever before. Sadly, from my perspective, this all seems to be worse in hospitals and often strongest in A&E where you need the most support and fastest action”*
		*“I have been discharged advising I'm not asthmatic only to be ventilated hours later! The arrogance can be astounding!”*
		*“Doctors saying take a breath or slow your breathing down, they really need to put themselves in the patient’s position when having an asthma attack”*
		*“GP services = don’t understand/care about how serious this condition can be”*
		*“Being told to just breathe and my oxygen stats are fine. I don't care what my oxygen is doing I just want to breathe”*
		*“Many healthcare professionals see difficult asthma as nothing more than difficult patients with asthma”*
		(What things help your asthma most?) “*Health professionals working with me and listening to my needs”*
	Others	*“I personally don’t think asthma is taken seriously enough, especially in schools”*
		*“The way others treat you, like asthma is like nothing more serious than a scraped knee”*
Costs	Prescription charges	*“Cost can be prohibitive”*
		*“Cost is a big issue. Prescription charges are ridiculous”*
Environmental factors and triggers	Weather	*“Living in Scotland the constant change in weather means I have to have 2 of each inhaler”*
		*“The environment......unfortunately you can't control the elements and they set asthma off”*
		*“The unpredictable weather always is barrier”*
		*“But a brisk day with some fresh wind is great! I don't feel like I'm struggling to breathe”*
	Allergens	*“Limited help from NHS* (National Health Service) *with allergen reduction”*
		*“Allergies....allergic to everything from food, animals, pollen, grass! The allergies are unknown so it makes controlling it hard”*
	Other triggers	*“Catching colds which can drop peak flow in a matter of 1 or 2 days which frequently leads to chest infection which can take doubled up inhalers, blue inhalers, prednisolone and at least 2 courses of antibiotics”*
		*“Brittle asthma difficult to control anything can set me off”*
		*“Not sure of all my triggers there are so many”*
Healthcare systems	Timely access to care	*“Slow replies and long wait times for appointments”*
		*“Sometimes slow to respond - I know what I need but still have to go through the bureaucracy to get it!”*
		*“It’s usually quicker to get an appointment with my GP to discuss my needs as opposed to the asthma nurse where waiting times are often several weeks”*
	Follow up and continuity	*“Different doses of prednisolone prescribed depending on which doctor I speak to within the same practice”*
		*“Lack of continuity of care when I see a GP at my local health centre”*
		*“Not getting the right follow up care with GP after asthma attack”*
		*“Lack of continuity of care from my GP as you never see the same one and have to tell your story every time”*
	Communication	*“Doctors don't read what my consultant writes”*
		*“They will not contact Royal Brompton, will nor read letters from RBH* (Royal Brompton Hospital)*”*
		*“Also delays between being seen at casualty and the info reaching health centre”*
	Obtaining medication	*“My GP keeps messing up my prescription, meaning I can't always get my inhaler and tablets for asthma when I run out as they refuse to sign the meds over due to communication error with dosage. I have also been told I can't have an inhaler at one point when I had run out because my hospital was the one who authorised the drug and not my GP”*
		*“Not getting my meds when I need them, which means I end up in hospital with a flare up”*
		*“I have to allow 3 days for a new prescription to be emailed”*
Lifestyle factors	Inconvenience/time	*“Remembering to take them* (inhalers) *in the evening before I go out. If I forget I have to come home early as I struggle to breathe”*
		*“I also use the small vent* (spacer device) *which needs washed out, it would be easier if an inhaler lasted all day as I do forget”*
		*“Self-management is also hard as life tends to take over sometimes, since I work in a hospital myself I know the importance but I wish it would just go away!”*
	Exercise	(What are the barriers to controlling your asthma?) *“Coming up with an appropriate exercise plan”*
		*“Little but often exercise learning what I can or can’t do without taking the inhaler first”*
	Diet	*“I once tried an anti-candida (low sugar) diet. reduced it by two thirds and had quite a lasting effect but difficult to maintain*”
		(What things help your asthma most?) *“Eating healthily and small portions”*
Medication	Efficacy	(What do you do when you have an asthma attack?) *“Take my inhaler (2puffs) and continue to do so until I feel improvement”*
		(What do you do when you have an asthma attack?) *“Take two puffs of my reliever inhaler. If still not better take a further two puffs one at a time every two minutes, for up to 10 puffs, or until symptoms have improved”*
		(What things help your asthma most?) *“Taking my medication regularly and when needed quickly”*
	Education	(What are the problems/issues you face in taking your regular asthma medication?) *“Knowing when to take brown pump”*
		*“Sometimes I can’t do it at all because the attack makes me unable to remember what do”*
	Side effects	*“My wife was on every tablet inhaler and even having xolair injections but the main problem she had was controlling her weight due to the steroids”*
		*“The steroid tablets for me are an issue with all the different side effects. Especially the weight issue when you are told you need to lose weight but know yourself that it is almost impossible to do this when you are taking inhalers with steroids plus the tablets*”
		“*Moon face, gaining weight, bad bones, mood swings, sleepless nights, mouth thrush”*
Self-care	Monitoring	(What do you do when you have an asthma attack?) *“Check my peak flow”*
		(What things help your asthma most?) *“Air alert service”*
	Personalised action plan	(What things help your asthma most?) *“Having an asthma attack plan and to know what to do if i have one i.e. keep calm”*
	‘Common sense’ measures	(What things help your asthma most?) *“Me knowing my limits and not pushing myself”*
		(What do you do when you have an asthma attack?) *“Sit down or stand and place my back against something”*
	Staying calm/breathing exercises	(What do you do when you have an asthma attack?) *“Practise good breathing techniques”*
		(What do you do when you have an asthma attack?) *“Slow deep breaths (as deep as I can manage)”*
		(What things help your asthma most?) *“Remaining calm when having an attack”*
Support	Healthcare professionals	(What things help your asthma most?) *“Having a very good GP who tries to understand and listen to me”*
	Others	(What things help your asthma most?) *“Having understanding people around”*
		(What do you do when you have an asthma attack?) *“Call my husband to stay near”*

### Attitudes and knowledge

Some respondents felt that attitudes of both healthcare professionals and family and friends can be a barrier to successful management of their asthma. Many commented that asthma can be trivialised, leading to problems such as delays in appropriate treatment or embarrassment taking medication in public:

“*Sometimes they* (healthcare professionals) *treat you as if asthma is not serious but I know that it can be as I have been in ICU* (intensive care unit) *with asthma numerous times”*

*“For me, personally, the main barrier is the attitude of people towards asthma - many of the general public see it as 'just' asthma and not a serious condition”*

Respondents also expressed doubts about the level of knowledge of the professionals caring for them. Some recognised that this may simply be a limitation of current medical research, while others felt that their healthcare providers ought to be better informed:

*“To be honest I think the doctors and scientists are finding it as hard as curing cancer”*

*“GP* (general practitioner) *does not know what to do anymore”*

(What are the barriers to controlling your asthma?) *“Uneducated GPs…”*

### Costs

Ten respondents raised issues around the cost of prescriptions, which impacts their ability to take their medication as prescribed and thus inhibits asthma control:

*“Cost of prescription. Sometimes I tend to miss evening doses of my inhaler…to make it last longer.”*

*“The cost of prescriptions. I have 5 separate medicines and that is expensive every month.”*

### Environmental factors and triggers

Respondents mentioned issues related to the weather, pollution, allergies and other triggers, often outside their control. These themes were cited both as barriers to asthma control, and in relation to the importance of recognising and reducing exposure for improved control:

*“I can't control all of my triggers like stress or the weather”*

*“At this moment in time as my allergies are so bad, I feel nothing is helping me”*

### Healthcare systems

Issues around getting the healthcare provision that people felt they needed was mentioned frequently. Respondents were particularly troubled by lack of timely access to appropriate care, while others noted that ready access to medical care could greatly facilitate their asthma management:

*“Waiting times. Busy phone lines. Sometimes it can take 10 attempts to get through to someone I need”*

*“My severe asthma consultants and their team are superb and look holistically at my problems and I can call to speak to an asthma nurse if I want or need some advice, my GP is also very cooperative”*

Within this theme, respondents also highlighted problems around continuity of care and communication between healthcare staff as negatively impacting on their asthma management:

*“Sometimes when I am not able to see my regular GP they do not understand as much about me.”*

*“Poor relationship between GP and specialist clinic”*

Conversely, several respondents mentioned that regular reviews can facilitate their control and empower them to self-manage their condition:

*“Regular asthma reviews with the doctor or nurse so I can monitor my asthma and be reassured I am doing everything right”*

Finally within this theme respondents mentioned difficulties associated with obtaining their asthma medication and the impact that this can have on their control:

*“My GP's receptionists don't seem to understand how prescription issues can be a huge worry for someone with 'just' asthma but simply not being able to access my repeat or having someone change it for no apparent reason are major issues.”*

### Lifestyle factors

Lifestyle factors could be both a barrier and a facilitator to asthma control. Respondents mentioned diet, exercise and the time or inconvenience associated with managing their asthma:

*“I want to exercise but find this is very difficult and results in uncontrolled asthma for a period of time”*

*“I am losing weight and that has helped with my breathing”*

*“Making sure I have 2 inhalers, 1 for home and 1 for work. That the one at work is accessible if I have no pockets as I work in a school”*

### Medication

Issues around medication use were mentioned by almost all respondents. Respondents almost universally reported that one of the first things they do during an acute attack is use their reliever medication:

*“Take salbutamol inhaler and if it's not quite clearing but only need a little more I'll take a puff of my* [combined inhaler] *and wait 15 minutes. If it's not clearing or too bad I use a nebuliser”*

*“It was my wife who suffered with brittle asthma and as her carer the first thing I did was get her on the nebuliser”*

Respondents also discussed their regular medication in terms of helping them to successfully manage their asthma day to day:

*“In terms of medication, getting the right combination can work wonders - more can sometimes be more but more can also sometimes give you a bit of your life back (even with side effects!)”*

However, a smaller number of respondents raised questions about the efficacy of their prescribed medication or the appropriateness of their therapeutic regimen:

*“It is no longer effective”*

*“Not sure I am on right inhalers”*

In addition, a few respondents mentioned that inadequate education about use of their medication could be a barrier to control:

(What are the problems/issues you face in taking your regular asthma medication?) *“Not been shown how to properly use them”*

Finally within this theme, respondents raised concerns about side effects of medication and for some this was a major barrier to asthma control:

*“My GP and consultant have always been very hot on making sure medications are taken correctly but I'm in the position that I'm on so much that I have a lot of side effects”.*

*“To have taken high dose prednisolone for over 28 years. Had hip replacement, problems with spine and neck developed reflux, epilepsy, diabetes, arthritis, high blood pressure, high cholesterol… Put on lots of weight”*

*“Dry powder inhalers difficult to use and give me a sore throat/tongue plus thrush”*

### Self-care

Respondents mentioned important self-care measures that they implement both during acute episodes and more generally to manage their condition day to day. This included ‘common-sense’ measures such as finding fresh air, sitting in a comfortable position and moving away from any possible triggers but also referring to written action plans and more formal self-monitoring:

(What do you do when you have an asthma attack?) *“Sit myself down on a chair and throw my arms over the chair back. Loosen any tight clothing”*

(What things help your asthma most?) *“I also refer to my written asthma plan regularly”*

(What things help your asthma most?) *“Keep an eye on peak flows”*

Within this theme, we also considered issues around stress and staying calm, both during an attack and as a longer term measure for improving asthma control. Such points were raised by respondents in their answers to several of the questions asked. The importance of keeping calm and controlled breathing was frequently mentioned, as was the risk of panicking during an acute attack:

*“Try to stay calm, sit down and try to breathe slowly”*

*“Stay calm, count as it helps slow breathing down”*

*“Sometimes I panic”*

*“Feel terrified”*

### Support

Finally, some respondents discussed the importance of the support, or lack of, that they receive to help them to manage their asthma. This included both support from healthcare professionals and others and in an acute and non-acute situation:

(What things help your asthma most?) *“The biggest thing is having support to deal with things not only when they deteriorate but also on a daily basis - asthma can take over your life but having the right support makes that easier to deal with.”*

(What things help your asthma most?) *“Support from medical staff and family”*

(What are the barriers to controlling your asthma?) *“Unsupportive medical staff”*

### Triangulation with workshop outcomes

We used the themes described above to inform the findings of the concurrent workshop. We present the research areas identified as priorities in Table 3, alongside the theme from the survey data to which they relate. Presented in bold are the topics which were considered amenable to being answered by a Cochrane Review. Some areas were already addressed by existing reviews. The ten new Cochrane Review topics were directly derived from the research priorities developed and ranked at the workshop and informed by the themes from this survey.

**Table 3:** Prioritised research topics with winked survey themes

Research question/topic	Relevant survey theme(s)
What is the most effective way of organising local asthma services to ensure all people with asthma can access information & support? (e.g. specialist nurses)	Healthcare systems
**Education about living with asthma (reducing social stigma) AND training to help children having asthma attacks for teachers. A) managing attacks/worsening symptoms; B) general awareness - "normalising"**	Attitudes and knowledge
	Support
What is the best way to ensure access to up-to-date, relevant information about regular treatments & how they work & ensuring appropriate understanding? (i.e. taking it when well) - for different populations	Healthcare systems
	Medication
**What is the effect of understanding your asthma attack & having a personalised asthma plan (based on what has worked before?)**	Self-care
**What do we know about the short-term and long-term effects of regular medication (e.g. steroids), how much is safe, when to 'step up' or 'step down'?**	Medication
What is the best way to achieve shared understanding between doctors & patients with acute asthma?	Attitudes and knowledge
	Healthcare systems
**Reasons for not taking medications (e.g. oral steroids) for patients are different to what doctors are thinking (oral steroids make me crazy; I can't sleep on steroids versus bone density)**	Medication
How do we work in partnership with patients & empower them to treat asthma alongside other conditions & consider how drugs & symptoms overlap & interact?	Attitudes and knowledge
	Healthcare systems
	Medication
**Asthma acute attack and anxiety: do the following help? Using a pulse oximeter (measure oxygen); a fast-track asthma service; phone application for monitoring asthma; being told nothing works**	Lifestyle factors
	Self-care
	Healthcare systems
**For teenagers & adults - does learning how to stay calm (breathing/yoga) help you to manage asthma attack?**	Lifestyle factors
	Self-care
What evidence-based self-help (e.g. diet, vitamin D, exercise) measures are useful in controlling long-term symptoms/asthma?	Lifestyle factors
Drs/consultants/nurses/other healthcare professionals talking to each other. More joined-up thinking (& practicalities - e.g. consultant advises treatment which GP then prescribes)	Healthcare systems

Bold type face: topics which were considered amenable to being answered by a Cochrane systematic review

## Discussion

### Principal findings

We found that the majority of respondents were experiencing important barriers to successful management of their asthma. Barriers largely related to their interaction with health services, including timely access to and continuity of care, attitudes and knowledge of healthcare staff and obtaining medication. Other barriers included lack of support, difficulty avoiding triggers and managing stress and panic.

Facilitators were less frequently mentioned but included regular follow-up, support from healthcare staff and others, efficacious medication and appropriate self-management strategies both during an acute attack and day-to-day.

We developed a list of priority Cochrane Reviews based on what was said by people affected by asthma in this survey and an asthma workshop. This demonstrates the real impact that people affected by asthma have on the research agenda of Cochrane Airways.

### Strength and weaknesses

The online nature of the survey ensured respondents were able to complete it in privacy and anonymously, likely leading to more honest responses. Their answers were not influenced by the ‘group effect’ in a focus group setting or by a person administering the survey. Questions were standardized for all respondents and were open-ended allowing free-text responses. As it was internet-administered, we obtained a large number of responses in a short time period with potentially wide geographical reach. There was no risk of researcher selection bias. Participation was voluntary and not incentivised in any way so there was no risk of coercion.

External validity and generalizability of the survey findings are limited by the anonymity and self-selection of participants; we cannot comment on how representative this sample is of people with asthma in the UK, nor can we put their responses in the context of asthma severity, age, gender or location. If we were to carry out a similar survey in the future we would seek to rectify this by requesting basic non-identifying demographic information such as age bracket, gender and number of attacks requiring medical attention in the last 12 months. This would help greatly to contextualise our findings.

Findings may also have been influenced by the time of year at which the survey was conducted, during the summer in the UK. The survey was conducted at this time because it was part of a wider prioritisation exercise which was ongoing in 2014. However, it would be prudent for similar future studies to invite participation at different time points through the year to ensure the seasonality of asthma symptoms for some people is not skewing the results. This would also allow researchers to look for trends and assess consistency of the responses given to enhance the reliability of the findings.

As the survey was advertised via resources aimed at supporting and educating people with asthma and their friends and families, it is possible that respondents may be generally more engaged with managing their asthma and not fully representative of those affected by asthma as a whole. Those who chose to respond may also have seen it as an opportunity to express either frustration or appreciation. This could potentially skew our findings and further limit generalizability. With appropriate ethics approval, future researchers could consider sending invitations to participate in the survey via a GP asthma register, which is more likely to capture a generalizable sample of people with asthma. However, it may still be those who are most engaged with the management of their condition who choose to respond.

In addition, the wording of the questions may have somewhat influenced the responses, for example, asking about ‘problems’ with healthcare may be more likely to elicit a negative response than simple asking a person to ‘describe’ their experiences of healthcare.

### Comparison with other studies

Many of the themes which emerged from our survey data are in line with existing studies, including a focus on side effects of drugs [12, 13]; inconvenient or unpleasant medication administration techniques; psychological aspects of managing a chronic condition [12]; personal decision making and self-management [13]; healthcare organisation; social environment; and costs [11]. In contrast, the effect that attitudes and knowledge of health professionals has on management of a medical condition, mentioned frequently by our respondents, does not seem to be prominently reported in the literature as an identified research priority.

## Conclusions

We report the findings of an online survey which formed part of a larger project which produced ten priority review topics for a programme of work at Cochrane Airways. The themes which emerged added credibility and dependability to the findings of the prioritisation workshop and the previous prioritisation activities undertaken by the group [5].

Taken alone, the findings offer a valuable insight into the challenges faced by individuals affected by asthma in the UK, and possibly further afield. However, external validity of our findings was limited by our lack of information about respondents. Future research surveys should perhaps endeavour to collect basic participant details including age, gender, self-reported asthma severity and county of residence.

Many of the barriers highlighted by respondents are problems with attitudes, knowledge and healthcare systems which may be difficult to address. However, some of the barriers may be more amenable to change. One example might involve general practices introducing systems that allow patients with asthma to request medication urgently, rather than having to wait the typical 24–48 hours for a repeat prescription and additional training of reception staff to respond appropriately to requests from a patient with asthma. Another example might be to ensure that all patients with asthma have a ‘named GP’ to maximise continuity of care. Practices could consider adding an ‘alert’ on a patient’s electronic notes that advises a same-day appointment should always be booked if requested. Better advertising by GPs and pharmacists of the pre-payment scheme for prescriptions [19] may alleviate some of the financial concerns expressed, as would including asthma on the prescription-charge exemption list [20].

Encouragingly, we received reports of good quality care which enabled respondents to manage their condition more effectively. Features of good quality care included regular medical reviews and continuity of care, support of professionals, friends and family, easy access to timely care in an emergency and a holistic approach to the person. Patients with asthma may want to consider choosing their GP with these points in mind.
